# Cardiomyocyte ionic currents in intact young and aged murine *Pgc-1β^−/−^* atrial preparations

**DOI:** 10.1016/j.mad.2017.11.016

**Published:** 2018-01

**Authors:** Haseeb Valli, Shiraz Ahmad, Anita Y. Jiang, Robert Smyth, Kamalan Jeevaratnam, Hugh R. Matthews, Christopher L.-H. Huang

**Affiliations:** a Physiological Laboratory, University of Cambridge, Downing Street, Cambridge CB2 3EG, United Kingdom; b Faculty of Health and Medical Sciences, University of Surrey, Guildford GU2 7AL, Surrey, United Kingdom; c PU-RCSI School of Medicine, Perdana University, 43400 Serdang, Selangor Darul Ehsan, Malaysia; d Department of Biochemistry, University of Cambridge, Tennis Court Road, Cambridge CB2 1QW, United Kingdom

**Keywords:** Na^+^ currents, K^+^ currents, Age-dependent arrhythmia, *Pgc-1β^−/−^*, Energetic deficiency

## Abstract

**Introduction:**

Recent studies reported that energetically deficient murine *Pgc-1β^−/−^* hearts replicate age-dependent atrial arrhythmic phenotypes associated with their corresponding clinical conditions, implicating action potential (AP) conduction slowing consequent upon reduced AP upstroke rates.

**Materials and methods:**

We tested a hypothesis implicating Na^+^ current alterations as a mechanism underlying these electrophysiological phenotypes. We applied loose patch-clamp techniques to intact young and aged, WT and *Pgc-1β^−/−^*, atrial cardiomyocyte preparations preserving their in vivo extracellular and intracellular conditions.

**Results and discussion:**

Depolarising steps activated typical voltage-dependent activating and inactivating inward (Na^+^) currents whose amplitude increased or decreased with the amplitudes of the activating, or preceding inactivating, steps. Maximum values of peak Na^+^ current were independently influenced by genotype but not age or interacting effects of genotype and age on two-way ANOVA. Neither genotype, nor age, whether independently or interactively, influenced voltages at half-maximal current, or steepness factors, for current activation and inactivation, or time constants for recovery from inactivation following repolarisation. In contrast, delayed outward (K^+^) currents showed similar activation and rectification properties through all experimental groups. These findings directly demonstrate and implicate reduced Na^+^ in contrast to unchanged K^+^ current, as a mechanism for slowed conduction causing atrial arrhythmogenicity in *Pgc-1β^−/−^* hearts.

## Highlights

• Energetically-deficient *Pgc-1β−/−* murine atria show age-dependent arrhythmia.
• Voltage clamp studies investigated their underlying membrane current changes.
• *Pgc-1β−/−* atria showed reduced inward Na^+^ currents with normal voltage-dependences.
• Outward repolarising K+ currents retained normal activation and rectification.
• A resulting slowed action potential conduction explains the arrhythmic phenotype.

## Graphical abstract

**Figure:**
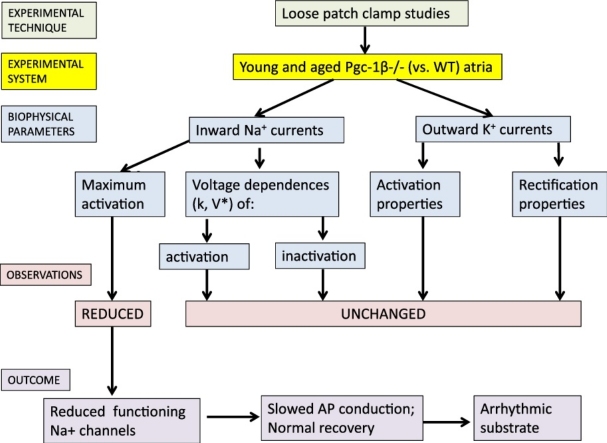

## 1 Introduction

Cardiac arrhythmias typically follow disruption of the normally coordinated activation and inactivation processes in successive ion channel species mediating cardiac action potential (AP) propagation, initiation and recovery and constitute a major clinical source of morbidity and mortality. Thus, atrial fibrillation (AF) is associated with five-fold increased risks of stroke and increased risks of all-cause mortality (Benjamin et al., 1998, Friberg et al., 2007, Chamberlain et al., 2015). Growing evidence implicates metabolic, particularly mitochondrial, dysfunction whether reflecting ageing itself (Sun et al., 2016) or age-dependent manifestations of metabolic syndrome including obesity (Bournat and Brown, 2010), insulin resistance (Patti and Corvera, 2010) and hypertension in the pathogenesis of AF (Dikalov and Ungvari, 2013). Animal AF models show abnormal mitochondrial structure and function (Morillo et al., 1995, Ausma et al., 1997). Cardiomyocyte mitochondria from AF patients show increased DNA damage (Tsuboi et al., 2001, Lin et al., 2003), structural abnormalities (Bukowska et al., 2008) and impaired function (Lin et al., 2003, Ad et al., 2005).

Cardiac arrhythmic mechanisms related to specific monogenic ion channel disorders have previously been studied in genetically modified murine models which provided valuable insights into contributions of particular channels to both arrhythmic triggering and the substrate ensuring persistence of the resulting arrhythmic disturbance (Huang, 2017). Thus, studies in *Scn5a^+/−^* hearts implicated compromised conduction velocities arising directly from loss of Na_v_1.5 function in not only ventricular but also atrial arrhythmic substrate in Brugada Syndrome (Guzadhur et al., 2012, Martin et al., 2012). The resulting slowed conduction in depolarising AP wavefronts promoted re-entrant circuit formation at the tissue electrophysiological level causing sustained arrhythmia. Reduced atrial conduction velocities, thus implicated in long term arrhythmic substrate (Park et al., 2009, Miyamoto et al., 2009) have been reported in early clinical AF (Zheng et al., 2017).

Murine models, particularly those deficient in peroxisome proliferator activated receptor-γ coactivator-1 (PGC-1) transcriptional coactivators also proved useful in biochemical studies of metabolic conditions. PGC-1α and PGC-1β are highly expressed in cardiac, brain and skeletal muscle, tissues with high oxidative capacity (Riehle and Abel, 2012). They are key regulators of mitochondrial mass and function (Lin et al., 2005, Finck and Kelly, 2006), increasing expression of nuclear and/or mitochondrial encoded proteins involved in the tricarboxylic acid cycle, fatty acid β–oxidation and oxidative phosphorylation (Arany et al., 2005). PGC-1 expression levels are reduced in obesity, insulin resistance, type II diabetes mellitus and ageing, in parallel with the associated mitochondrial dysfunction (Mootha et al., 2003, Leone and Kelly, 2011, Dillon et al., 2012).

Recent electrocardiographic studies reported age-dependent slowing of myocardial action potential (AP) conduction in *Pgc-1β^−/−^* hearts (Ahmad et al., 2017). Intracellular recording studies then reported age-dependent *atrial* arrhythmic phenotypes associated with the AP conduction abnormalities in intact Langendorff perfused *Pgc-1β^−/−^* hearts (Valli et al., 2017) agreeing with previous reports on their pro-arrhythmic *ventricular* phenotypes (Gurung et al., 2011). The slowed conduction in *Pgc-1β^−/−^* atria was attributed to reduced maximum action potential upstroke rates, (d*V*/d*t*)_max_, relative to those in WT (Valli et al., 2017). (d*V*/d*t*)_max_ has been correlated with both peak Na^+^ currents (*I*_Na_), responsible for the rising, activation phase of the propagating action potential (AP) and its conduction velocity in both skeletal and cardiac muscle cells (Usher-Smith et al., 2006, Fraser et al., 2011). Such young and aged, WT and *Pgc-1β^−/−^* atria contrastingly showed indistinguishable resting potentials, as maintained by outward K^+^ currents.

These associations suggest hypotheses attributing pro-arrhythmic changes in *Pgc-1β^−/−^* atria to compromised Na^+^ channel (Na_v_1.5) function reducing voltage-dependent Na^+^ currents (I_Na_). Previous evidence at the cellular as opposed to tissue levels had suggested that in addition to compromised ATP provision, disrupted cardiomyocyte mitochondrial activity increases reactive oxygen species (ROS) production (Fosset et al., 1988, Faivre and Findlay, 1990) and perturbs cytosolic NAD^+^/NADH, both implicated in I_Na_ reductions (Liu et al., 2009), rescued by the mitochondrial ROS scavenger mitoTEMPO (Liu et al., 2010). In addition, recent studies reported altered Ca^2+^ homeostasis manifest as abnormal diastolic Ca^2+^ transients in *Pgc-1β^−/−^* cardiomyocytes (Gurung et al., 2011). In addition to driving pro-arrhythmic triggering delayed after-depolarisations, such cytosolic [Ca^2+^] elevations could potentially modify Na_v_1.5 properties through either direct or indirect Ca^2+^ actions at its C-terminal region (Mori et al., 2000, Wingo et al., 2004) or calmodulin kinase II-phosphorylatable sites in its DI-II linker (Mori et al., 2000, Wagner et al., 2011, Grandi and Herren, 2014). Slowed AP conduction with reduced (d*V*/d*t*)_max_ similarly associated with reduced I_Na_ have been reported in other pro-arrhythmic murine, *RyR2*-P2328S/P2328S, cardiomyocyte models similarly showing abnormal Ca^2+^ handling (Zhang et al., 2013)_._ This was attributed to both reduced expression (Ning et al., 2016) and acute effects of altered [Ca^2+^]_i_ upon. Na_V_1.5 function (King et al., 2013b, King et al., 2013c).

The present experiments explored whether the previously reported pro-arrhythmic *Pgc-1β^−/−^*atrial phenotype (Valli et al., 2017) is similarly accompanied by altered I_Na_. The loose patch technique employed for voltage-clamping in intact, young and aged, WT and *Pgc-1β^−/−^*, atrial cardiomyocytes apposes an electrode containing extracellular solution against intact cardiomyocyte surface membrane without accessing intracellular space. It therefore measured ion currents under conditions of unperturbed extracellular [Na^+^] and intracellular Ca^2+^ homeostasis (Almers et al., 1983a, Stühmer et al., 1983, King et al., 2013b). This contrasts with the cardiomyocyte isolation and intracellular Ca^2+^ chelation required with conventional whole-cell patch clamp techniques (Lei et al., 2005, Gurung et al., 2011, Martin et al., 2012). Recent studies involving reversible manipulations of extracellular [Na^+^] had identified early inward currents in response to step depolarisations measured under loose patch clamp with Na^+^ currents responsible for AP conduction and the maximum upstroke rate, (d*V*/d*t*)_max_, of the cardiac action potential (King et al., 2013b). The present experiments assessed activation, inactivation, and recovery from inactivation of depolarising inward currents attributable to Na_v_1.5, comparing these with corresponding activation and rectification properties of repolarising outward K^+^ currents.

## 2 Materials and methods

### 2.1 Animals and ethical approval

This research has been regulated under the Animals (Scientific Procedures) Act 1986 Amendment Regulations 2012 following ethical review by the University of Cambridge Animal Welfare and Ethical Review Body (AWERB). C57/B6 mice maintained in an animal facility under 12-h light/dark cycles at a maintained (21 °C) temperature, were fed sterile chow (RM3 Maintenance Diet, SDS, Witham, Essex, UK) and provided with free access to water, bedding and environmental stimuli. *Pgc-1β^−/−^* mice were generated using the triple LoxP targeting vector previously described (Lelliott et al., 2006). Four experimental groups were studied: young WT (age: 12–16 weeks), young *Pgc-1β^−/−^* (12–16 weeks), aged WT and aged *Pgc-1β^−/−^* (>52 weeks). Mice were administered 200 IU of unfractionated heparin (Sigma-Aldrich, Poole, UK) intraperitoneally before sacrifice by cervical dislocation (Schedule 1; Animals (Scientific Procedures) Act 1986).

### 2.2 Experimental preparations

Chemicals were obtained from Sigma-Aldrich (Poole, UK) unless otherwise stated. Immediately following sacrifice, the heart was excised and transferred into ice-cold Krebs–Henseleit (KH) solution: (mM) NaCl, 108; NaHCO_3_, 25; KCl, 4.0; KH_2_PO_4,_ 1.2; MgCl_2,_ 1.0; CaCl_2,_ 1.8; glucose, 10; and Na-pyruvate, 2.0; pH adjusted to 7.4 and bubbled with 95% O_2_/5% CO_2_ (British Oxygen Company, Manchester, UK). The aorta was cannulated with a trimmed and filed 21G hypodermic needle, and secured onto the cannula with an aneurysm clip and 5-0 braided silk suture. The heart was perfused retrogradely in a Langendorff system under constant flow (2.1 ml/min) by a Watson-Marlow (Falmouth, UK) peristaltic pump with 75 ml of a modified KH solution containing 10 mM 2,3-butanedione monoxime (BDM) and 10 μM blebbistatin (Cayman Chemical Company, Ann Arbor, Michigan, USA) (to give a KH-BDM/blebbistatin solution) to electromechanically uncouple the heart. The heart was then immediately transferred into ice-cold KH-BDM/blebbistatin solution. The atria were dissected from the rest of the heart, mounted onto Sylgard (Dow Chemical Company, Staines, UK) and placed in a bath containing filtered KH buffer solution. The latter was thermostatically maintained at 27 °C through all the experimental procedures performed.

### 2.3 Loose patch clamp recording

Pipettes were pulled from borosilicate glass capillaries (GC150-10 Harvard Apparatus, Cambourne, Cambridge, UK) using a Flaming/Brown micropipette puller (model P-97, Sutter Instrument Co. Novato, CA, USA). Pipettes were mounted and fractured with a diamond knife at 250× magnification under a microscope with a calibrated eyepiece graticule, Applying a transverse force to the distal tip of the pipette gave a fracture perpendicular to the main micropipette axis. Selected pipettes were fire polished using an electrically heated nichrome filament under visual guidance at 400× magnification. The pipette tips were then bent to make a ∼45° angle with the pipette shaft. This permitted them to approach the membrane vertically when mounted on the recording amplifier headstage. Maximum internal pipette tip diameters were measured at 1000× magnification.

All pipettes had diameters 28–32 μm following polishing. Their distal ends were filled with KH buffer and mounted onto the pipette holder connected to the headstage. Ag/AgCl electrodes maintained electrical connections to the organ bath and pipette. The pipette was lowered onto the membrane surface. Gentle suction was applied through an air-filled line connected to the pipette holder using a syringe to induce seal formation around the membrane patch. Voltage-clamp steps were delivered under computer control relative to resting membrane potential (RMP). The loose patch clamp configuration results in larger leakage currents than conventional patch clamp owing to relatively low seal resistances. A custom-built amplifier compensated for most of the leakage current, series resistance errors and displacement currents through the pipette capacitance. These gave typical values of electrode resistances of 0.2–0.3 MΩ, and typical ‘loose-patch’ seal resistances of ∼1.0 MΩ comparable to previous studies using this technique (Stühmer et al., 1983). Residual leakage and capacitative currents were then corrected for using reference records from subsequent P/4 control protocols applying steps of the opposite sign relative to the test steps, with amplitudes scaled down by a factor of 4 as fully described previously (Almers et al., 1983a, Almers et al., 1983b). Once established all patches were subject to the complete set of pulse procedures bearing on either inward or outward current activation. Patch consistency was monitored through repeat calibrations of leakage current, series resistance and pipette capacitance (Stühmer et al., 1983). We studied totals of 25 patches from male and 21 patches from female WT, and 24 patches from male and 17 patches from female *Pgc-1β^−/^*^−^ hearts, but found no significant differences (P > 0.05) between maximum Na^+^ currents in preparations from male and female hearts. We accordingly grouped such data together when examining effects of the remaining factors of age and genotype. Curve-fitting procedures employed in analysis of data used the fitting algorithm in the open source R programming language. Statistical analysis of results employed two way ANOVA to the experimental groups of young and aged, WT and *Pgc-1β^−/^*^−^ to test for independent or interacting effects of age and/or genotype on any significant differences, This was followed by Tukey’s honestly significant difference testing to detect pairwise differences.

## 3 Results

**Fig. 1:**
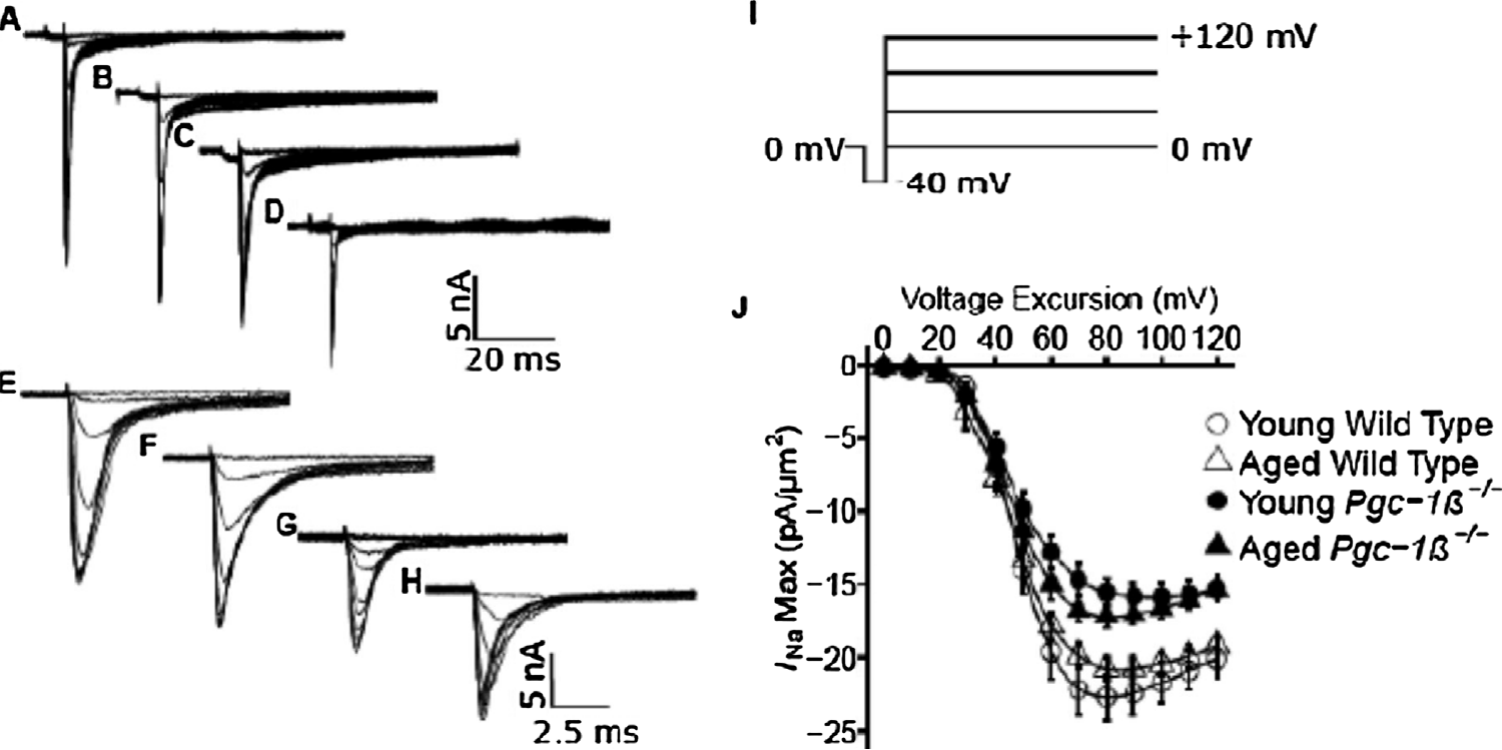
Activation properties shown by voltage-dependent inward Na^+^ currents. Typical records in young (A, C, E, G) and aged (B, D, F, H) wild-type (WT; A, B, E, F) and *Pgc-1β^−/−^* atria (C, D, G, H), at slow (A–D) and fast (E–G) time bases in response to (I) activation pulse protocols beginning from the resting membrane potential (RMP). A prepulse (duration 5 ms) was made 5 ms into the recording period to (RMP − 40 mV). This was followed by successively larger depolarising test voltage steps increased in +10 mV increments up to (RMP + 120 mV). (J) Peak currents, I_NaMax,_ plotted against voltage excursion for young (circles) and aged (triangles), wild-type (clear symbols) and *Pgc-1β^−/−^* atria (filled symbols).

**Fig. 2:**
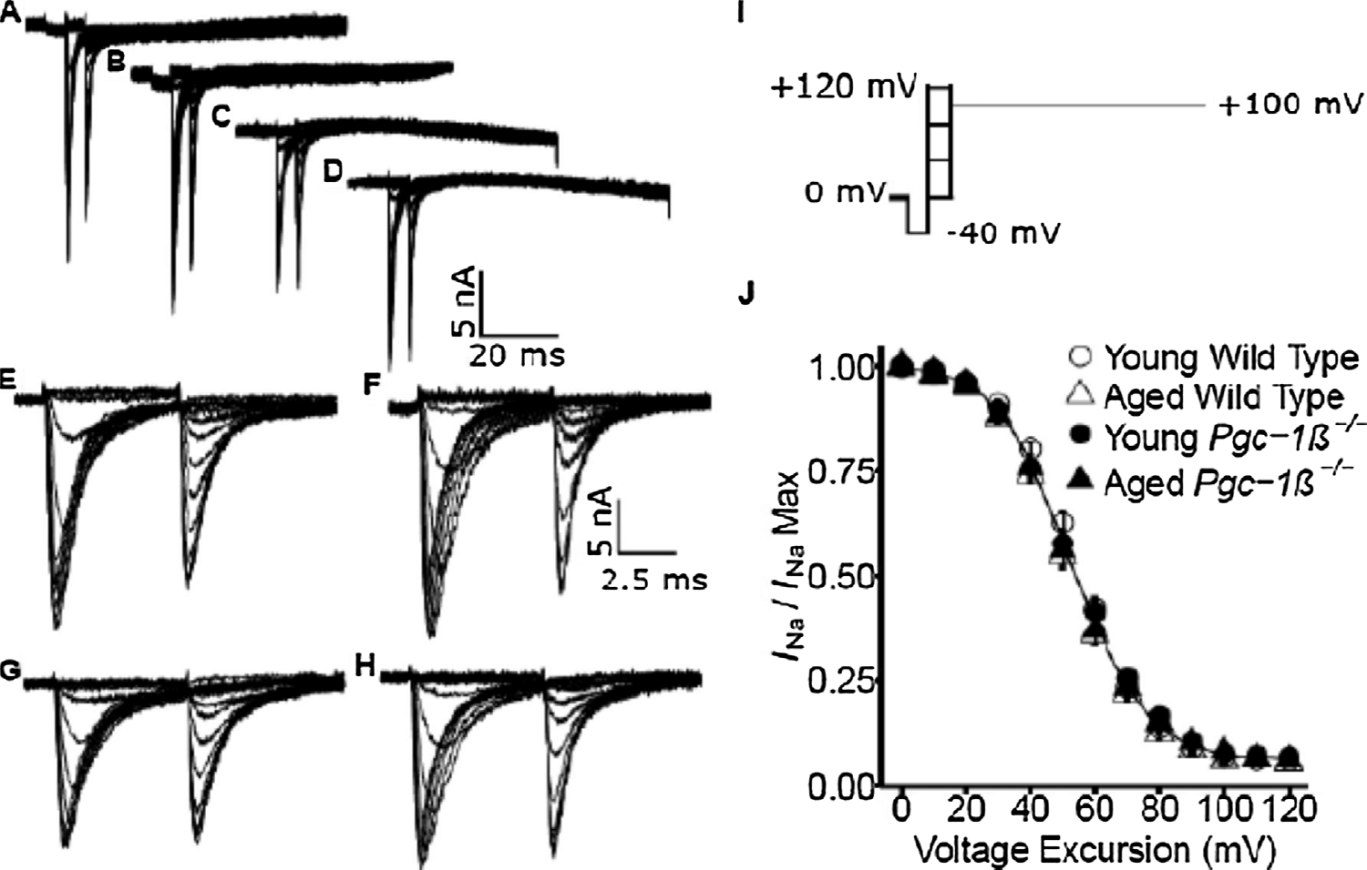
Investigation of inactivation properties shown by voltage-dependent inward Na^+^ currents. Typical records in young (A, C, E, G) and aged (B, D, F, H) wild-type (WT; A, B, E, F) and *Pgc-1β^−/−^* atria (C, D, G, H), at slow (A–D) and fast (E–G) time bases in response to inactivation pulse protocols applied from the resting membrane potential (RMP). (I) In the pulse protocol, a prepulse (duration 5 ms) was made 5 ms into the recording period to (RMP − 40 mV). This was followed by successively larger depolarising conditioning voltage steps increased in +10 mV increments up to (RMP + 120 mV) of 5 ms duration. Finally the voltage was stepped to a constant test level of (RMP + 100 mV), and the resulting Na^+^ currents quantified to investigate the inactivation brought about by the preceding conditioning step. (J) Peak currents I_NaMax_ plotted against voltage excursion for the conditioning voltage step in young (circles) and aged (triangles), wild-type (clear symbols) and *Pgc-1β^−/−^* atria (filled symbols).

**Fig. 3:**
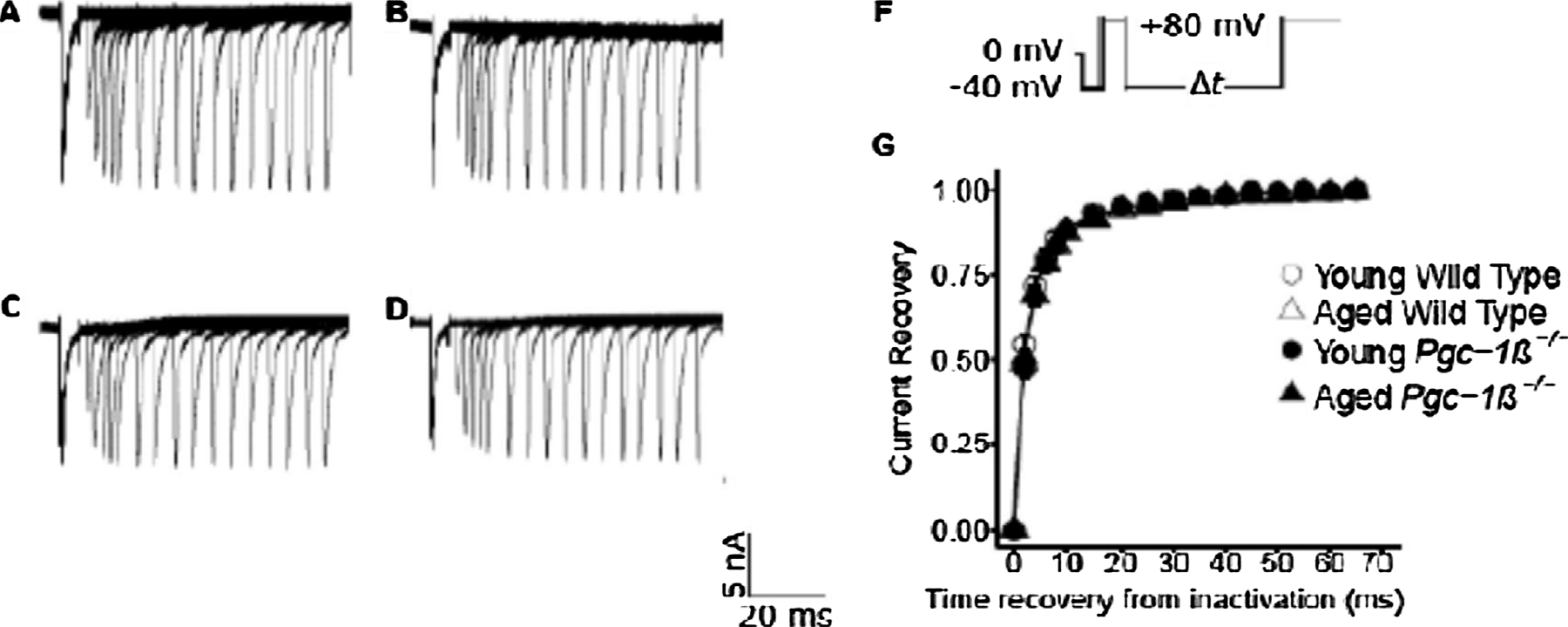
Currents illustrating Na^+^ channel recovery from inactivation. Records in young (A, C) and aged (B, D) wild-type (WT; A, B) and *Pgc-1β*^*−/−*^ atria (C, D). The pulse protocols (F) first held membrane voltages at RMP for 1 ms from the beginning of the recording period, then imposed a hyperpolarising prepulse to (RMP − 40) mV prior to the 5 ms duration P1 conditioning step to (RMP + 80 mV). The subsequent 5 ms duration test steps to (RMP + 80 mV) were imposed after different time intervals, Δ*T*, The latter were successively increased between 2 and 75 ms, in 2 ms increments for the first 5 sweeps and in 5 ms increments for the remainder of the 16 successive sweeps making up the protocol. (G) Plots of the recovery of peak I_Na_ against time intervening between termination of the conditioning and imposition of the test pulse.

**Fig. 4:**
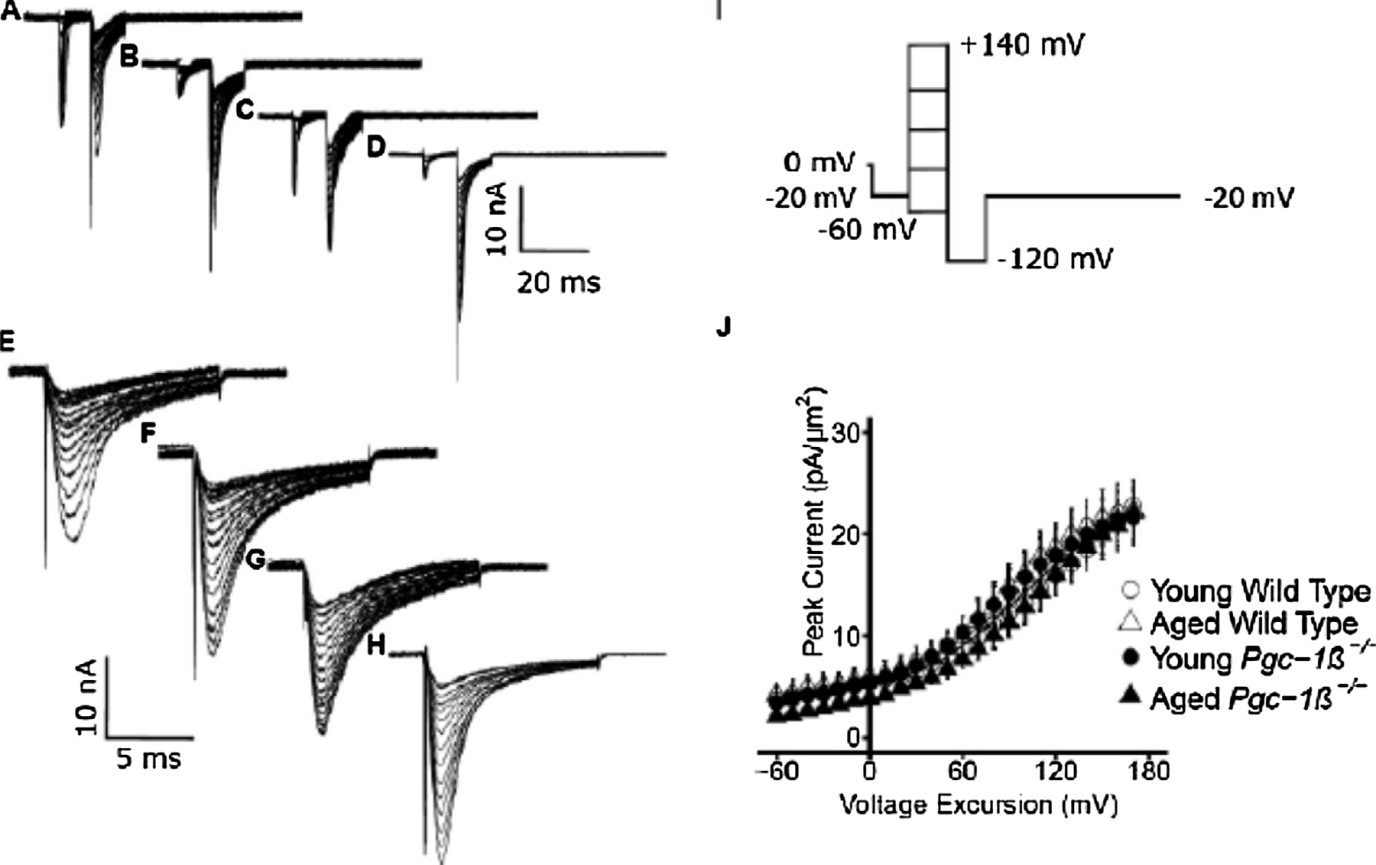
K^+^ current activation properties reflected in tail currents. Records from young (A, C, E, G) and aged (B, D, F, H) wild-type (WT; A, B, E, F) and *Pgc-1β^−/−^* atria (C, D, G, H), at slow (A–D) and fast (E–G) time bases. Pulse procedures (I) first applied a voltage step between 1 and 10 ms following the beginning of the recording period from RMP to (RMP − 20 mV). The following 10 ms duration test steps were made to voltages between (RMP − 60 mV) to (RMP + 170 mV) incremented in 10 mV steps through the 24 sweeps investigated. The final 10 ms duration hyperpolarising step to (RMP − 120 mV) that preceded final restoration of the membrane potential to (RMP − 20 mV) gave tail currents reflecting (J) the preceding K^+^ current activation, plotted against voltage excursion in the young (circles) and aged (triangles) WT (open symbols) and *Pgc-1β*^*−/−*^ atria (filled symbols).

**Fig. 5:**
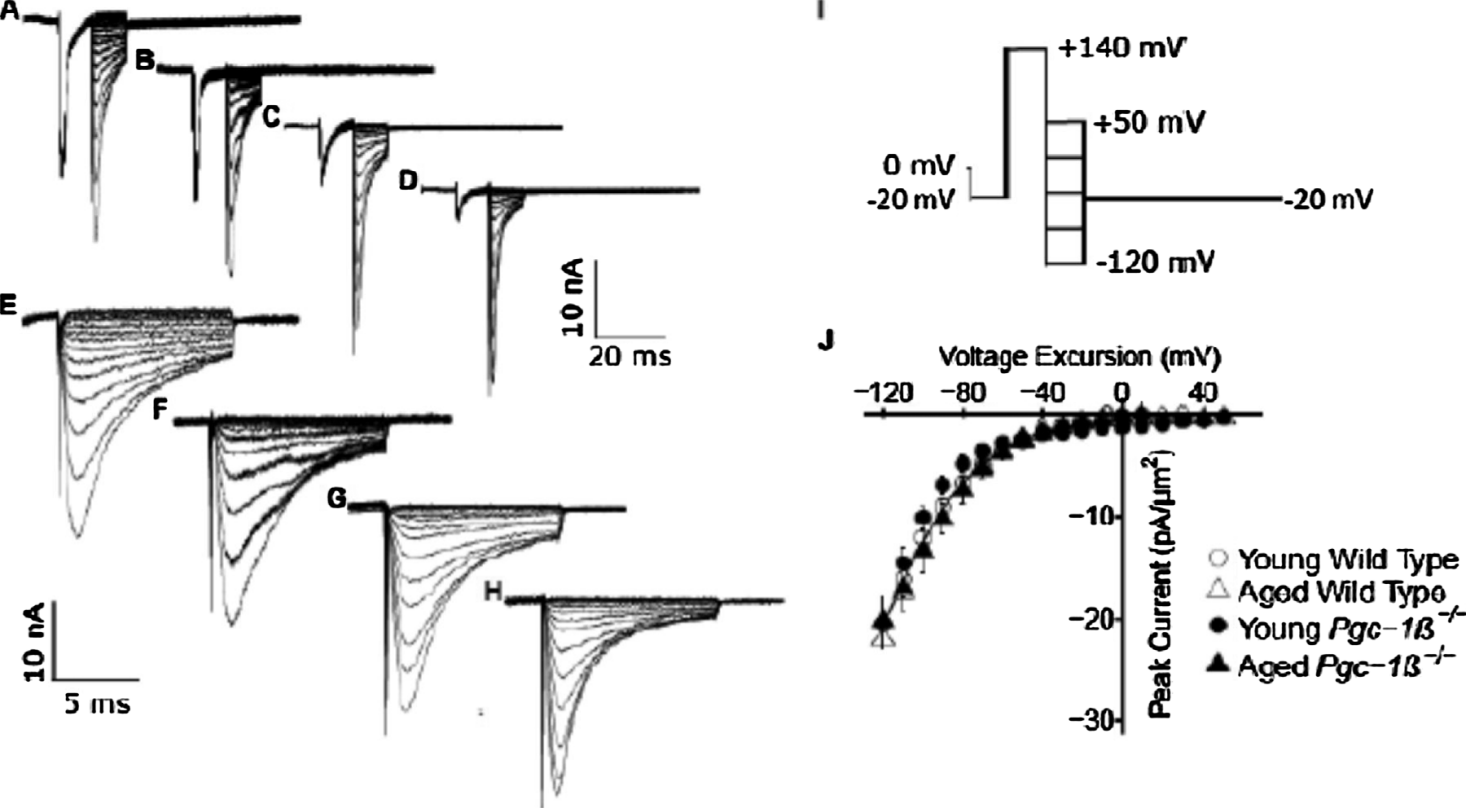
K^+^ current rectification properties reflected in tail currents. Typical records from young (A, C, E, G) and aged (B, D, F, H) wild-type (WT; A, B, E, F) and *Pgc-1β^−/−^* atria (C, D, G, H), at slow (A–D) and fast (E–G) time bases. The pulse procedure (I) first applied a voltage step between 1 and 10 ms after commencement of the recording period from RMP to (RMP − 20 mV). A following 10 ms duration test step was then made to a fixed voltage of (RMP + 140 mV). The following step to a varying voltages between (RMP − 120 mV) to (RMP + 50 mV) provided tail currents which could be plotted to obtain (J) instantaneous current-voltage relationships reflecting rectification properties of the activated channel in the young (circles) and aged (triangles) WT (open symbols) and *Pgc-1β^−/−^* atria (filled symbols). The protocol ended by restoration of the membrane potential to (RMP − 20 mV).

Patches obtained following seal formation were subject to pulse protocols that could completely characterise the properties of either voltage dependent inward or outward currents, each within 30 s. This made the likelihood of effects due to rundown minimal. In any case, measured currents remained consistent, when protocols were repeated over longer time intervals (∼15–20 min) in a number of control experiments. Finally, any given pulse protocol was always completed without altering the patch seal. This made differences between results attributable to changes in the patch over prolonged intervals such as bleb formation unlikely (Milton and Caldwell, 1990). As adopted in previous reports utilizing this technique, membrane potentials are expressed as voltage excursions relative to the RMP in the protocols illustrated in Fig. 1, Fig. 2, Fig. 3, Fig. 4, Fig. 5 (Almers et al., 1983a, Almers et al., 1983b). Thus the loose patch configuration differs from that involved in intracellular microelectrode or conventional cell-attached tight patch recording in leaving the intracellular space unperturbed. Instead, it applies a patch electrode on, forming a seal with, the external face of an intact surface membrane of the cell, initially at its resting membrane potential (RMP). It then applies voltage steps on the *extracellular* surface of the resulting membrane patch within the seal. Accordingly *positive* and *negative* voltage steps applied through the pipette respectively *hyperpolarise* and *depolarise* the membrane potential from its RMP.

### 3.1 Currents reflecting atrial inward Na^+^ current activation

Fig. 1 illustrates results obtained from the isolated atrial preparations. These explored activation properties of inward Na^+^ currents in young (panels A, C; E, G) and aged (B, D, F, H) wild-type (WT; A, B, E, F) and *Pgc-1β^−/^*^−^ atria (C, D, G, H). Results are shown both at slow (A-D) and rapid (E-H) timebases demonstrating full decays in and regions of the trace displaying the detailed kinetics of the currents respectively. The pulse protocols that investigated the voltage dependence of Na^+^ current activation (panel I) first held the cells at their RMP for 5 ms from the beginning of the recording period to establish an initial steady resting baseline. This was followed by a 5-ms duration prepulse to a hyperpolarised voltage, *V*_0_ = (RMP − 40 mV), that was expected to fall within a voltage range in which both Na^+^ channel activation and inactivation would be minimal. This thus both removed any residual Na^+^ current inactivation and standardised the initial activation state of the Na^+^ channels within the patch. This was followed by imposition of the depolarising test steps which became successively larger through the 13 successive recorded sweeps. They were made to voltages successively incremented between *V*_1_ = RMP to (RMP + 120) mV in +10 mV increments. The voltage steps extended to the end of the record length which was of total duration 80 ms. The currents were corrected for residual leakage by a P/4 protocol to give the family of records reflecting the voltage dependence of Na^+^ channel activation in which inward currents are represented as downward, negative deflections.

Traces typically began with a consistent small upward deflection in response to the −40 mV prepulse (A–D). The subsequent voltage steps to level *V*_1_ yielded a family of inward currents characteristic of Na^+^ currents, initially increasing with time to a peak value that increased with more positive *V*_1_. This was followed by a decay reflecting channel inactivation whose extent and kinetics was similarly determined by the voltage *V*_1_ (E–H). However, although young and aged atria showed similar current magnitudes, *Pgc-1β^−/−^* atria showed consistently reduced Na^+^ current amplitudes compared to WT.

### 3.2 Currents reflecting atrial Na^+^ current inactivation

In contrast, Fig. 2 shows records from protocols exploring atrial Na^+^ current inactivation properties in young (panels A, C; E, G) and aged (B, D, F, H), wild-type (WT; A, B, E, F) and *Pgc-1β^−/−^* atria (C, D, G, H). As previously, cells were first held at the RMP for 5 ms to establish an initial steady resting baseline. This was followed by application of a 5-ms duration prepulse to *V*_0_ = (RMP − 40) mV. This thus removed any residual Na^+^ current inactivation and standardised the initial activation state of Na^+^ channels within the patch, prior to the voltage steps that followed. This was followed by depolarising steps to conditioning voltages that were varied with the 13 successive sweeps between *V*_1_ = RMP to (RMP + 120 mV) in +10 mV increments. This conditioning step would elicit a voltage-dependent Na^+^ current activation as similarly achieved in the previous protocols that had been used to study Na^+^ channel activation properties. However, maintaining the imposed depolarisation then produced a Na^+^ current decline reflecting a Na^+^ channel inactivation whose extent would be dependent upon the prepulse voltage excursion *V*_1_. Following a 5 ms interval following imposition of the conditioning step, a test step was applied to a fixed voltage *V*_2_ = (RMP + 100 mV) and this extended to the end of the record length (panel I). This yielded a second set of current responses (Fig. 2A–H) that gave peak Na^+^ currents corresponding to a constant level of channel activation, that were however modified by the prior channel *inactivation* brought about by the conditioning voltage excursion to *V*_1_. This accordingly gave families of Na^+^ currents that *decreased* in amplitude with the previous inactivation brought about by the increasing *V*_1_. Thus, only channels spared inactivation by the prepulse to *V*_1_ would contribute currents in response to the step to the fixed voltage *V*_2_. Again, young and aged atria showed similar current magnitudes, but *Pgc-1β^−/−^* atria showed consistently reduced Na^+^ current amplitudes compared to WT.

### 3.3 Voltage dependences of atrial Na^+^ current activation

Figs. 1J and 2J respectively illustrate voltage-dependences of atrial Na^+^ current activation and inactivation for young (circles) and aged (triangles), WT (open symbols) and *Pgc-1β^−/^*^−^ atria (filled symbols), plotting peak Na^+^ current (means ± standard error of the mean (SEM)) against voltage excursion to *V*_1_. The quantifications of current-voltage and inactivation curves expressed the observed currents (nA) as current densities (pA/μm^2^) using the formula:

$$Current\;density=\frac{1000\;x\;current}{\left(\pi \;x\;pipette\;radius^{2}\right)}$$

In activation plots, peak inward Na^+^ current increased with the amplitudes of the depolarising steps exceeding +10 mV in size to a maximum value at a voltage excursion around +80 mV. They then decreased with further depolarisation as expected with approach of *V*_1_ towards the Na^+^ current reversal potential. Peak Na^+^ currents, *I* *=* *I*_NaMax_, reflecting activation properties, were empirically related to the activating voltage *V* = *V*_1_ by a Boltzmann function: *I* = *I*_max_/{1 + exp(*V* − V*/*k*)} where *I*_max_ is maximum current, V* is voltage at half-maximal current, and *k* is the steepness factor (Chadda et al., 2017).

Young and aged *Pgc-1β^−/−^* then showed similar maximum values of peak atrial inward currents (−16.97 ± 0.88 (n = 20) and −18.07 ± 0.89 (n = 21) pA/μm^2^ respectively) (Fig. 1J). These were reduced compared to values in both young (−23.93 ± 1.52 (n = 24) pA/μm^2^) and aged WT (−21.53 ± 0.84 (n = 22) pA/μm^2^). Two-way ANOVA demonstrated differences attributable to independent effects of genotype (F = 22.28; p = 0.95 × 10^−5^), but not age (F = 0.46; p = 0.50), or interacting effects of age and genotype (F = 2.48; p = 0.12). Post hoc Tukey tests demonstrated significant differences between young WT and young *Pgc-1β^−/−^* (p = 0.00016), young WT and aged *Pgc-1β*^*−/−*^ (p = 0.0016) and aged WT and young *Pgc-1β*^*−/−*^ atria (p = 0.027).

In contrast, V* values were similar amongst young (49.22 ± 1.92 (n = 24) mV) and aged WT (46.04 ± 1.65 (n = 22) mV), and young (48.94 ± 2.92 (n = 20) mV) and aged *Pgc-1β^−/−^* atria (46.31 ± 1.95 (n = 21) mV). Thus two-way ANOVA demonstrated no independent effects of either genotype (F = 0.003; p = 0.959) or age (F = 1.90; p = 0.172), nor did it show interacting effects of age and genotype (F = 0.016; p = 0.898). Values of *k* were also similar amongst young (6.21 ± 0.41 (n = 24) mV) and aged WT (7.18 ± 0.45 (n = 22) mV), and young (7.49 ± 0.42 (n = 20) mV) and aged *Pgc-1β^−/−^* (6.85 ± 0.44 (n = 21) mV). The two-way ANOVA demonstrated no independent effects of either genotype (F = 1.33; p = 0.25) or age (F = 0.24; p = 0.63), nor did it show interacting effects of age and genotype (F = 3.51; p = 0.065).

### 3.4 Voltage dependences of atrial Na^+^ current inactivation

In the inactivation plots, peak inward currents observed in response to depolarising steps to a constant voltage decreased with more positive prepulse voltages *V*_1_, reflecting inactivation increasing with increasing degrees of prior depolarisation (Fig. 2J). The peak currents reflecting inactivation properties, normalised to their maximum value observed with fully polarised prepulse voltages, were similarly empirically related to the inactivating voltage *V* = *V*_1_ by a Boltzmann function: *I* = *I*_max_ {1 − [1/{1 + exp(*V* − V*/*k*)]}. These gave similar values for V* amongst young (54.75 ± 0.98 (n = 24) mV) and aged WT (51.44 ± 1.14 (n = 22) mV), and young (52.98 ± 1.24 (n = 20) mV) and aged *Pgc-1β*^*−/−*^ atria (51.94 ± 1.71 (n = 21) mV). Two way ANOVA demonstrated no independent effects of either genotype (F = 0.32; p = 0.57) or age (F = 3.09; p = 0.082), nor did it reveal interacting effects of age and genotype (F = 0.79; p = 0.38). Similar *k* values occurred amongst young (10.62 ± 0.35 (n = 24) mV) and aged WT (11.15 ± 0.35 (n = 22) mV), and young (11.61 ± 0.25 (n = 20) mV) and aged *Pgc-1β*^*−/−*^ atria (10.65 ± 0.60 (n = 21) mV). Two-way ANOVA demonstrated no independent effects of either genotype (F = 0.35; p = 0.55) or age (F = 0.18; p = 0.67), nor did it reveal interacting effects of age and genotype (F = 3.33; p = 0.072).

Together these findings demonstrated effects of genotype but not age upon maximum peak Na^+^ currents *I*_max_, but not voltages at half maximal current V* or steepness factors, *k*, of Boltzmann functions describing either their activation or inactivation properties.

### 3.5 Time courses of atrial Na^+^ channel recovery from inactivation

Fig. 3(A–D) shows typical currents obtained from young (A, C) and aged (B, C), WT (A, B) and *Pgc-1β*^*−/−*^ atria (C, D) reflecting timecourses of Na^+^ current recovery from inactivation following restoration of the baseline voltage after an initial conditioning depolarising step to a fixed voltage. The pulse protocols (Fig. 3F) held the membrane voltages at the RMP for 1 ms from the beginning of the recording period, then imposed a hyperpolarising prepulse to voltage *V*_0_ = (RMP − 40 mV) for 4 ms to establish consistent baseline levels of Na^+^ current inactivation as in the previous protocols. A 5 ms-duration P1 conditioning step between *V*_0_ and *V*_1_ = (RMP + 80 mV) then elicited a Na^+^ current activation followed by its inactivation decay. Subsequent depolarising 5 ms-duration P2 steps to voltage *V*_3_ = (RMP + 80 mV) were imposed after different time intervals, Δ*T*, that were successively increased between 2 and 75 ms, in 2 ms increments for the first 5 sweeps and in 5 ms increments for the remainder of the 16 successive sweeps making up the protocol. The P2 steps elicited a Na^+^ current activation whose peak amplitude reflected the Na^+^ current recovery from inactivation with time, when normalised to corresponding values in the P1 step. Fits of time constants, τ, to the exponential function *I* = *I*_max_(1 − exp(−Δ*T*/*τ*)) describing this recovery (Fig. 3G) gave similar values of τ in young (3.44 ± 0.39 (n = 24) ms) and aged WT (3.70 ± 0.30 (n = 22) ms), and young (3.88 ± 0.31 (n = 20) ms) and aged *Pgc-1β^−/−^* (3.64 ± 0.30 (n = 21) ms) that did not reflect any independent effects of either genotype (F = 0.334; p = 0.565) or age (F = 0.007; p = 0.932), or of interacting effects of age and genotype (F = 0.561; p = 0.456) with two-way ANOVA.

### 3.6 Voltage dependences of atrial outward K^+^ current activation

These findings contrast with the similar voltage dependences and rectification properties of voltage-dependent total outward, K^+^, current amongst the experimental groups studied. The present experiments thus investigated such outward currents in murine atrial preparations using the loose-patch technique for the first time. Fig. 4 displays typical currents obtained from pulse procedures comparing voltage dependences of overall K^+^ current activation in young (Fig. 4A, C, E, G) and aged (Fig. 4B, D, F, H), WT (Fig. 4A, B, E, F) and *Pgc-1β^−/−^* (Fig. 4B, D, F, H) atria at slow (A-D) sweep speeds encompassing the entire record as well as rapid timebases encompassing the current tail reflecting K^+^ channel activation at the end of the preceding depolarising step (E-H).

The pulse procedure (Panel I) involved an initial imposition of a voltage step from the RMP to (RMP − 20 mV) between 1 and 10 ms from the beginning of the recording period to establish an initial steady resting state of channels within the patch. This was followed by a 10 ms duration test step made to a series of test voltages between (RMP − 60 mV) to (RMP + 170 mV) to explore the voltage dependence of K^+^ channel activation. The latter was incremented in 10 mV steps through the 24 sweeps that were investigated. These activation steps resulted in an initial inward Na^+^ channel activation, followed by its inactivation. However, this was succeeded in some traces by the gradual development of a small outward current reflecting activation of a rectified voltage dependent K^+^ current whose extent was dependent upon the voltage of the test step. This was then followed by a hyperpolarising step of duration 10 ms to a fixed post pulse voltage of (RMP − 120 mV) that would thereby apply a fixed driving voltage upon any K^+^ current flow through channels opened by the preceding test step. In the resulting family of K^+^ tail currents, their maximum magnitudes would therefore be determined by the instantaneous conductance reflecting the preceding K^+^ current activation. The pulse protocol ended with final restoration of the membrane potential to (RMP − 20 mV).

Fig. 4J plots typical activation current-voltage curves for the young (circles) and aged (triangles) WT (open symbols) and *Pgc-1β*^*−/−*^ (filled symbols) atrial preparations investigated. They demonstrated close to superimposable plots enclosing areas with the abscissa in which there were neither independent (F = 0.39; P = 0.54 and F = 0.079; P = 0.79 respectively) nor interacting (F = 1.75; P = 0.20) effects of either genotype or age.

### 3.7 Rectification properties of outward K^+^ currents in loose patched atrial preparations

The corresponding K^+^ current rectification properties were investigated by a pulse procedure similarly imposing an initial voltage step between 1 and 10 ms into the recording period from RMP to (RMP − 20 mV). However, the succeeding 10 ms duration test step was then made to a fixed voltage of (RMP + 140 mV) to achieve a specific level of K^+^ current activation. This was followed by a further voltage step to a range of voltages between (RMP–120 mV) to (RMP + 50 mV) in order to derive the instantaneous current-voltage relationship reflecting the rectification properties of the activated channel (Fig. 5I). Fig. 5(A–H) shows typical tail currents suggesting little or no difference in instantaneous current amplitudes between experimental groups.

Fig. 5J plots typical instantaneous current-voltage curves for young (circles) and aged (triangles) WT (open symbols) and *Pgc-1β*^*−/−*^ atria (filled symbols) demonstrating close to superimposable plots enclosing areas with the abscissa in which there were neither independent (F = 0.043; P = 0.84 and F = 0.97; P = 0.33 respectively) nor interacting (F = 0.005; P = 0.95) effects of either genotype or age.

## 4 Discussion

Increasing evidence implicates metabolic, particularly mitochondrial, dysfunction, a recognised feature of both ageing (Sun et al., 2016) and age-related metabolic disorders (Patti and Corvera, 2010, Bournat and Brown, 2010, Dikalov and Ungvari, 2013), in the pathogenesis of atrial fibrillation (Menezes et al., 2013), in both animal models (Morillo et al., 1995, Ausma et al., 1997) and clinical situations (Lin et al., 2003, Ad et al., 2005, Bukowska et al., 2008). The present studies examining accompanying alterations in electrophysiological function at the level of ion channel properties were prompted by recent reports describing electrophysiological pro-arrhythmic phenotypes at the tissue level in murine *Pgc-1β^−/−^* hearts (Valli et al., 2017) consequently deficient in this key mitochondrial regulator involved in the tricarboxylic acid cycle, fatty acid β–oxidation and oxidative phosphorylation (Arany et al., 2005, Lin et al., 2005, Finck and Kelly, 2006). The pro-arrhythmic phenotypes progressed with age to extents accentuated by the *Pgc-1β*^*−/−*^ as opposed to the WT genotype. These were accompanied by slowed AP conduction and compromised maximum action potential (AP) depolarization rates, (d*V*/d*t*)_max_ despite normal effective refractory periods and baseline action potential durations. These features together with an accompanying age-dependent fibrotic change, also accelerated in *Pgc-1β*^−/−^ relative to WT atria, could potentially furnish arrhythmic substrate. Reduced atrial conduction velocities have previously been reported in early clinical AF (Zheng et al., 2017) and to contribute to substrate for its long term maintenance (Park et al., 2009, Miyamoto et al., 2009).

At the tissue level, AP conduction depends upon local circuit currents generated by the rate of action potential depolarization (d*V*/d*t*)_max_ whose spread are in turn modified by membrane capacitance and cytosolic resistance, but for which previous studies correlated (d*V*/d*t*)_max_ with peak Na^+^ currents (*I*_Na_) (Jeevaratnam et al., 2011, King et al., 2013a). The recent studies accordingly suggested a hypothesis implicating the *Pgc-1β*^−/−^ as opposed to WT genotype, independently of age, in Na^+^ current reductions, but implicating both genotype and age in fibrotic changes that would additionally compromise local circuit currents propagating the resulting action potential activity. The consequent reductions in conduction velocity would then result in atrial pro-arrhythmic effects, as previously suggested for some canine AF models (Gaspo et al., 1997). *SCN5A* gene variants leading to reduced cardiac Na^+^ channel function have similarly been implicated in increased AF risks both in clinical situations (Olson et al., 2005, Darbar et al., 2008) and experimental studies in genetically modified *Scn5a^+/−^* murine hearts (Sabir et al., 2008, Kalin et al., 2010, Martin et al., 2011a, Martin et al., 2011b, Huang, 2017).

The present experiments applied a loose patch clamp method, which detects transmembrane current flowing into an extracellular electrode apposed to the membrane surface of cardiomyocytes within intact atrial tissue preparations (King et al., 2013b, Salvage et al., 2015, Ning et al., 2016). It thus avoids cytosolic disruptions that would follow the cell isolation and intracellular Ca^2+^ chelation required by conventional whole-cell patch-clamp recordings (Lei et al., 2005, Martin et al., 2012). It also allowed employment of in vivo rather than reduced extracellular [Na^+^] levels thereby sparing Na^+^-Ca^2+^ exchange processes. Ion currents were thus studied in atrial preparations under conditions similar to those employed in the previous reports on atrial arrhythmic phenotypes, and their associated changes in conduction velocity and (d*V*/d*t*)_max_ (Valli et al., 2017). Finally, previous reports had identified early inward currents obtained with this technique with Na^+^ currents mediating action potential (AP) conduction and upstroke (King et al., 2013b). The loose patch clamp technique has thus not been employed to study other inward, such as Ca^2+^, currents in detail. However, this may reflect the nature of the skeletal muscle and murine atrial preparations studied to date. These are associated with small Ca^2+^ relative to Na^+^ inward current contributions following activation by depolarising steps (Huang, 2017).

The loose patch clamp experiments demonstrated a voltage dependent activation of inward currents consisting of increases to a peak current followed by an inactivation decay giving a time course and a dependence upon the amplitude of progressively larger depolarising steps characteristic of Na^+^ currents in all the, young and aged, WT and *Pgc-1β^−/−^* atria studied. However, the presence of a *Pgc-1β^−/−^* genotype specifically resulted in a reduction in the peak Na^+^ current, without either independent or interacting influences of age. The remaining Na^+^ current characteristics in the form of either voltage at half maximum current, V*, or the steepness, *k,* of the Na^+^ current activation characteristics derived from the current-voltage curves were unaffected by either age or genotype. Imposition of steps to a fixed depolarised voltage level from a range of prepulse voltages similarly elicited currents rising to a peak followed by decay. The peaks now declined in amplitude with depolarising prepulse levels, reflecting the resulting voltage-dependent inactivation they would produce. However, inactivation curves constructed from plotting such peak currents against prepulse level yielded similar inactivation functions, as reflected in the similar V* or *k* values derived from voltage dependences of inactivation obtained in all four experimental groups. Finally, the specific differences in maximum Na^+^ current took place against indistinguishable outward repolarising, K^+^, current characteristics between groups. These first investigated voltage dependences of K^+^ current activation in response to pulse procedures employing a range of test steps, followed by hyperpolarising steps to a fixed voltage in order to assess the current tails reflecting the preceding activation. Conversely K^+^ current rectification properties were investigated imposing fixed voltage step to produce a constant level of activation. Accordingly, the succeeding steps to varying voltages then permitted open channel rectification properties to be explored. Both experiments yielded similar atrial currents from all four experimental groups, which accordingly yielded closely concordant activation and instantaneous current-voltage curves.

Together these findings thus demonstrate a possible mechanism for the genotypically-related variations in arrhythmic phenotype, with their accompanying reductions in AP conduction velocity and peak AP upstroke rates (d*V*/d*t*)_max_ in *Pgc-1β^−/−^* atria. They attribute these to reductions in maximum Na^+^ currents against a constant background of outward K^+^ current characteristics. They fulfil predictions at the level of intact atria, from previous studies at the cellular level reporting that metabolic stress potentially alters Na^+^ currents. This could take place through effects on Na^+^ channel activity of increased production of reactive oxygen species (ROS) or compromised NAD^+^/NADH ratios, effects rescued by the mitochondrial ROS scavenger mitoTEMPO and NAD^+^ restoration respectively (Liu et al., 2010, Gomes et al., 2013). *Pgc-1β^−/−^* cardiomyocytes also showed evidence for abnormal Ca^2+^ homeostasis (Gurung et al., 2011), in common with murine *RyR2*-P2328S atrial myocytes (Goddard et al., 2008), which similarly showed parallel AP conduction velocity and Na^+^ current reductions (King et al., 2013b). These were attributed to both *chronically* downregulated Na_v_1.5 expression (King et al., 2013b, Ning et al., 2016) as well as acute (King et al., 2013b, King et al., 2013c, Zhang et al., 2013) and potentially reversible loss of Na_v_1.5 function (Knollmann et al., 2001, Salvage et al., 2015, Salvage et al., 2017). The recent findings associating *Pgc-1β^−/−^* with increased rather than decreased *SCN5A* expression would be consistent with the latter mechanism involving direct effects of altered cytosolic [Ca^2+^] upon Na_v_1.5 function (Tan et al., 2002, Aiba et al., 2010, Ashpole et al., 2012). Such interactions could involve Ca^2+^-binding at the Na^+^ channel C-terminal region, either directly at an EF hand motif (Wingo et al., 2004) or indirectly through an IQ domain sensitive to calmodulin/calmodulin kinase II (Mori et al., 2000). There are also multiple phosphorylatable sites in the Na^+^ channel DI-II linker region including serines 516 and 571, and threonine 594 targeted by calmodulin kinase II (CaMKII) (Mori et al., 2000, Wagner et al., 2011, Grandi and Herren, 2014). Certainly previous studies have reported that elevations or sequestration of intracellular [Ca^2+^] respectively reduced or restored Na^+^ currents and (d*V*/d*t*)_max_ in WT cardiomyocytes in vitro (Casini et al., 2009).

## Conflicts of interest

None declared.
